# A real-world study of PARP inhibitors in 75 patients with platinum-sensitive recurrent ovarian cancer from China

**DOI:** 10.3389/fonc.2023.1300199

**Published:** 2024-01-08

**Authors:** Jinghong Chen, Mengpei Zhang, Kemin Li, Yuanqiong Duan, Xiaojuan Lin, Lan Zhong, Qintong Li, Rutie Yin

**Affiliations:** ^1^ Department of Obstetrics and Gynecology, West China Second University Hospital, Sichuan University, Chengdu, Sichuan, China; ^2^ Key Laboratory of Birth Defects and Related Diseases of Women and Children, Ministry of Education, Sichuan University, Chengdu, Sichuan, China

**Keywords:** PARP inhibitor, platinum-sensitive recurrent ovarian cancer, real-world study, progression-free survival, safety

## Abstract

**Objective:**

The aim of this study is to assess the efficacy and safety of poly (ADP-ribose) polymerase inhibitor (PARPi) as a maintenance therapy for patients with platinum-sensitive recurrent epithelial ovarian cancer (PSROC) at the largest center of gynecologic oncology in Western China.

**Patients and methods:**

The efficacy of PARPi was evaluated by progression-free survival (PFS) and overall survival (OS) in this real-world single-center retrospective cohort study conducted at West China Second University Hospital. The safety of PARPi was assessed using Common Terminology Criteria for Adverse Events Version 5.0.

**Results:**

In this study, we included a total of 75 eligible patients, of which 54 (72.0%) received olaparib and 21 (28.0%) received niraparib. Among these patients, 24 (32.0%) had breast cancer susceptibility gene (BRCA) mutations, 27 (36.0%) achieved complete response after their last platinum-based therapy, and 22 (29.3%) had previously received ≥3^rd^-line chemotherapy. The median progression-free survival (mPFS) was 19.1 months (95% CI 8.5-29.7), and the median overall survival (mOS) had not been reached. Log-rank analysis revealed that age (<65 years old V.S. ≥65 years old) and previous lines of chemotherapy (2^nd^-line V.S. 3^rd^-line V.S. ≥4^th^-line) were associated with prolonged PFS (*P <*0.05). However, multivariate COX regression analysis did not identify any independent factors associated with prognosis (*P >*0.05). The most common grade≥3 adverse events in the olaparib group were anemia, thrombocytopenia, and leukopenia, while in the niraparib group, they were anemia and thrombocytopenia.

**Conclusion:**

This study confirmed that olaparib and niraparib are effective and tolerate for PSROC in real-world settings. At the follow-up endpoint, no independent prognostic factor associated with prolonged PFS was identified.

## 1 Introduction

Ovarian cancer is the third most common female reproductive system malignancy. There were 313,959 new cases of ovarian cancer all around the world in 2020, including 55,342 new cases in China, accounting for 17.62% of the global new cases. A total of 207,252 deaths due to ovarian cancer in the world in 2020, including 37,519 cases in China, accounting for 18.10% of the global total (1). The onset of most patients is insidious, 70% of whom are diagnosed at an advanced stage while 70% relapse within 2-3 years, and the 5-year survival rate is only 30-40%. For patients with newly diagnosed advanced ovarian cancer, initial treatment is particularly crucial in comprehensive management. Maintenance therapy plays a significant role in overall management for ovarian cancer. Poly ADP ribose polymerase inhibitors (PARPi) has astounded the world time and time again with its maturing clinical data (2). Multiple large randomized controlled trials (RCTs) such as SOLO-1 (3), PAOLA-1 (4), PRIMA (5), and PRIME (6) studies have confirmed the curative effect of first-line maintenance therapy for advanced ovarian cancer. The population of SOLO1 trial was limited to BRCA-m patients, while BRCA-wt population was studied in the PRIMA and PRIME trials. They reported that niraparib maintenance therapy provided different degree of benefit in the first-line maintenance treatment of advanced ovarian cancer in the general population (5, 6). The PAOLA-1 study showed that in the HRD-positive population, OS was longer with olaparib plus bevacizumab (HR 0.62, 95% CI 0.45-0.85) (4).

In recent years, PARPi has become a standard treatment for patients with platinum-sensitive recurrent epithelial ovarian cancer (PSROC). The SOLO-2 study (7) revealed a 70% reduction in the risk of disease progression or death (HR=0.30, 95% CI 0.22-0.41) in PSROC patients treated with olaparib. The L-MOCA study (8) demonstrated that after a follow-up of 15.5 months, the median progression-free survival (mPFS) in the overall population, BRCA-mutation (BRCA-m) group, and BRCA wild-type (BRCA-wt) group were 16.1 months, 21.2 months, and 11.0 months, respectively. This study is the first to illustrate the efficacy of olaparib in the PSROC population among Asian individuals, regardless of BRCA mutation status. The NORA study (9) primarily focused on individualized starting doses for Chinese patients with PSROC. In the overall population, the group treated with niraparib demonstrated a 68% reduction in the risk of disease progression or death (HR=0.32, 95% CI 0.23-0.45). Among the gBRCA-m group, the niraparib group showed a 78% reduction in the risk of disease progression or death (HR=0.22, 95% CI 0.12-0.39). In the non-gBRCA-m subgroup, the niraparib group exhibited a 60% reduction in the risk of disease progression or death (HR=0.40, 95% CI 0.26-0.61). These findings highlight the significant impact of niraparib treatment across different patient subgroups. The updated OS data presented at the European Society for Medical Oncology (ESMO) Congress in 2022 revealed that, following the implementation of inverse probability weighting, the niraparib group exhibited a 30.8% reduction in the risk of disease progression or death compared to the placebo group (HR=0.692, 95% CI 0.446-1.074) in the overall population. In the gBRCA-m group, the niraparib group did not reach the mOS (HR=0.882, 95% CI 0.387-2.011). Notably, within the non-gBRCA-m population, the mOS for the niraparib group amounted to 43.1 months, marking a substantial 10.5 months extension compared to the placebo group (HR=0.624, 95%CI 0.368-1.056) (10). These large RCTs have laid a solid foundation for clinical diagnosis and treatment. However, these studies strictly adhere to specified inclusion criteria and treatment protocols, which effectively minimize bias but also result in discrepancies from real-world clinical scenarios (11). Real-world studies have better external validity which are essential to assess the benefit of new drugs in real clinical practice (12). Nevertheless, there is a lack of such real-world studies on PARPi, especially limited data based on the Chinese population. Consequently, the aim of this study was to evaluate the real-world clinical data from patients with PSROC who were administered PARPi as maintenance therapy and identify factors associated with long-term benefits to accumulate more clinical experience in PARPi maintenance therapy for patients with PSROC.

## 2 Patients and methods

### 2.1 Patients and inclusion criteria

The study, conducted in accordance with the principles of the Declaration of Helsinki and the guidelines of the International Conference on Harmonization of Good Clinical Practice, was approved by the Ethics Committee of West China Second University Hospital (approval number: 20220129). As a result of the retrospective design and anonymous data collection of this study, informed consent from the patients was not required.

The clinicopathological data of patients with PSROC treated with PARPi as maintenance therapy after recurrence were collected from August 1, 2018 to September 31, 2022 at the West China Second University Hospital. The inclusion criteria were as follows: (1) age ≥ 18 years old. (2) pathologically confirmed epithelial ovarian cancer, fallopian tube cancer or primary peritoneal cancer with complete clinical and pathological data. (3) patients who achieved complete response (CR) or partial response (PR) after the last platinum-based chemotherapy. (4) patients receiving PARPi for maintenance therapy after platinum-sensitive relapse. Patients who missed important clinical data or declined to follow up were excluded.

### 2.2 Data collection

Clinical and pathological data collection was conducted to build a real-world database using Microsoft Excel. The basic information of PSROC patients was extracted from the information systems of West China Second University Hospital, Sichuan University (including the Hospital Information System [HIS], laboratory information system, and Picture Archiving and Communication System [PACS]). Patients who met the inclusion and exclusion criteria were selected for the study. The patient-related information collected includes the following: (1) Baseline information: age, body mass index (BMI), comorbidities (hypertension, diabetes, thyroid dysfunction, chronic hepatitis B virus infection, etc.), family history, BRCA gene mutation status, initial treatment, and the number of previous lines of platinum-based chemotherapy. (2) Surgical related data: surgical outcome, postoperative pathological diagnosis, International Federation of Gynecology and Obstetrics (FIGO) 2014 staging. (3) Postoperative treatment status: first-line chemotherapy status (chemotherapy regimen, course of treatment, completion time of chemotherapy, response to chemotherapy), recurrence status (platinum-sensitive recurrence/platinum-resistant recurrence, chemotherapy regimen, course of treatment, completion time of chemotherapy, response to chemotherapy), maintenance treatment (CA125 baseline level before medication, CT/MRI before medication, medication time, starting dose, medication cycle, drug interruption, reduction, discontinuation and reasons, disease progression and time to progression, treatment after progression, and overall survival time). Missing information was supplemented by telephone follow-up or face-to-face inquiries (if alive and accessible).

### 2.3 Outcomes

The outcome of primary debulking surgery (PDS) or interval debulking surgery (IDS) was assessed based on the postoperative residual lesion size records and imaging data. The classification of the residual disease is defined as R0 for no visible residual lesions after surgical treatment, R1 for postoperative residual lesions ≤ 1 cm, and R2 for postoperative residual lesions > 1 cm. The response to chemotherapy was evaluated with Response Evaluation Criteria in Solid Tumors (RECIST) version 1.1 (13), which categorizes responses as CR, PR, stable disease (SD), or progressive disease (PD) (13). CR is defined as the disappearance of all target lesions, with the short axis of all pathological lymph nodes reduced to <10mm (13). PR indicates a reduction of the sum of target lesion diameters by at least 30% compared with the baseline level (13). SD lies between PR and PD, signifying neither sufficient shrinkage to qualify for PR nor an increase in lesion size to qualify for PD (13). Lastly, PD is marked by a relative increase of at least 20% in the diameter sum of all measured target lesions, and an increase in the absolute value of the diameter sum of at least 5 mm. Additionally, the appearance of one or more new lesions is also considered as part of the classification for PD (13). The efficacy was assessed by PFS and OS. PFS was defined as the period from the initiation of PARPi to radiographic progression according to RECIST version 1.1 (13), death from any cause, or study cutoff. OS was defined as the time from the start of PARPi treatment to death from any cause or study cutoff. The safety of PARPi was evaluated using the Common Terminology Criteria for Adverse Events Version 5.0, (CTCAE5.0) (14), as stipulated by the National Cancer Institute of the United States in 2017. Maintenance therapy after relapse refers to the continuation of treatment after achieving CR or PR following secondary cytoreduction (SCR) or the most recent platinum-based chemotherapy for PSROC patients. It aims to prolong the time to subsequent relapse and lessen associated risk. PSROC is defined as the time between receiving platinum-based chemotherapy and tumor recurrence and progression exceeding 6 months (6, 15, 16). Furthermore, the duration of the platinum-free interval (PFI) ranging from 6 and 12 months is termed as partial platinum-sensitive recurrence, while a PFI of more than 12 months is classified as complete platinum-sensitive recurrence (17).

### 2.4 Follow-up

This real-world study aimed to gather information about the patient’s living status, including the progression of the disease, instances of mortality and the causes of death. Furthermore, the study collected data on adverse events (AEs) experienced after medication, such as the specific AE terms, the highest CTCAE grade reported, treatment measures employed for AEs, as well as actions taken with regards to PARPi, such as reduction, interruption, and discontinuation. The study utilized various channels for data collection, including telephone, outpatient clinic visits, WeChat groups, and QQ groups. The follow-up endpoint is recurrence, progression, death, or the study cut-off date, which is December 1, 2022.

### 2.5 Statistical analysis

The statistical analysis was performed with SPSS version 25.0 software. For continuous variables that followed a normal distribution, they were presented as mean ± standard deviation (mean ± SD), and independent sample t-tests were used for group comparisons. If the variables did not follow a normal distribution, they were expressed as median (Q1, Q3), and group comparisons were performed using the Kruskal-Wallis test. Categorical variables were presented as counts (n) and percentages (%), and group comparisons were conducted using the Chi-square test (χ2). Additionally, survival curves were generated using GraphPad Prism 8.0.1 software. The median follow-up time was calculated using the reverse Kaplan-Meier method. A Log-rank univariate analysis was performed to evaluate factors associated with PFS for patients. Factors with a significance level of *P*<0.05 in the univariate analysis were included in the multivariate Cox regression analysis. A significance level of *P*<0.05 was used to define statistically significant differences.

## 3 Results

### 3.1 Baseline characteristics

**Figure 1:**
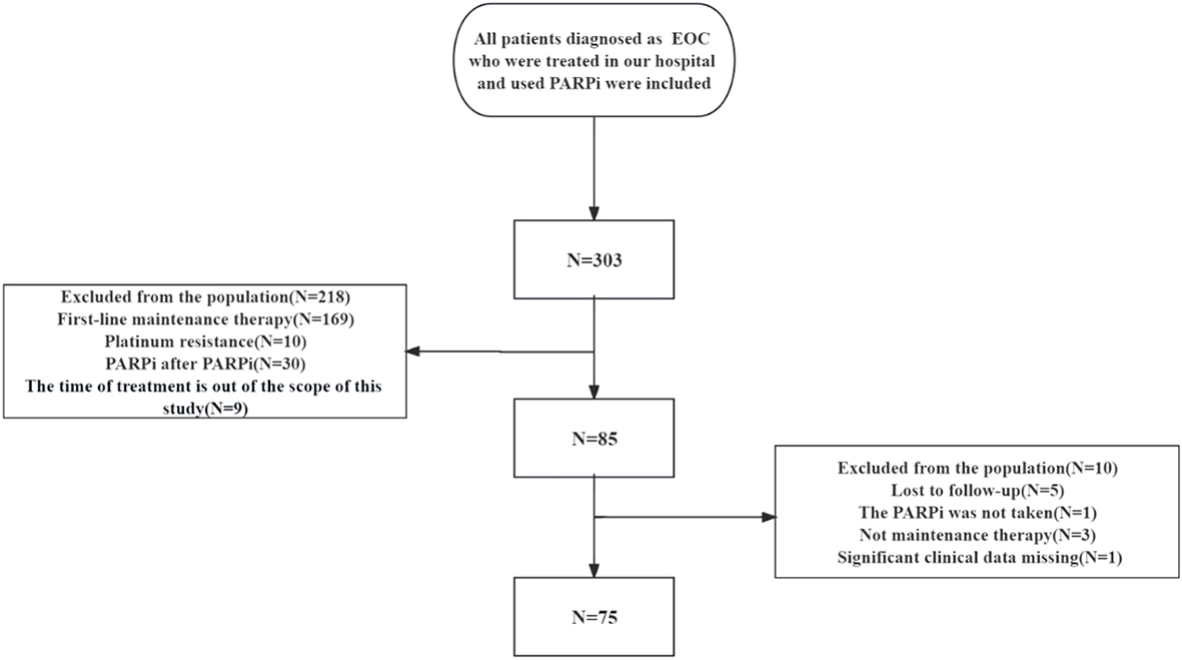
Enrollment flow diagram.

In this study, a total of 75 eligible patients were enrolled, with 54 (72.0%) receiving olaparib and 21 (28.0%) receiving niraparib as indicated in **Figure 1** and **Table 1**. Among these patients, 24 patients (32.0%) were found to carry BRCA-m, while 5 patients refused genetic testing due to economic reasons. 27 patients (27/75, 36.0%) had received neoadjuvant chemotherapy (NACT) in the past. After the primary surgery, 29 patients (38.7%) had no residual lesions, and 27 patients (36%) achieved R1. Additionally, 11 patients (14.7%) were unaware of any residual lesions after PDS/IDS. Among them, 9 had prior surgeries at different medical facilities, and 2 lacked information concerning residual lesions from their surgical records. Moreover, among 75 patients, 9 cases (12.0%) received SCR after PSR, all achieving R0 status. After the last platinum-based chemotherapy, 27 out of 75 patients (36.0%) achieved CR, while 48 patients (64.0%) achieved PR. Among the cohort, 22 (29.3%) had previously received 3rd-line or more lines of chemotherapy and 29 (38.7%) had experienced partial PSR (PFI 6-12m). It is important to note that the baseline characteristics indicated a balanced and comparable distribution of characteristics between the olaparib and niraparib groups (P>0.05).

**Table 1:** Clinicopathological characteristic of PSROC patients.

Clinical characteristics	Olaparib (N=54)	Niraparib (N=21)	Statistics	*P*
Age (mean ± SD, year)	52.6 ± 9.2	52.2 ± 6.8	–	0.763
BMI (median (Q1,Q3),kg/m^2^)	22.5 (21.4-23.6)	21.8 (20.6-23.5)	t=0.402	0.689
Complication, n (%)			χ2 = 0.151	0.697
Yes	18 (33.3)	8 (38.1)		
No	36 (66.7)	13 (61.9)		
Family history, n (%)			χ2 = 0.158	0.691
Yes	18 (33.3)	6 (28.6)		
No	36 (66.7)	15 (71.4)		
BRCA gene, n (%)			χ2 = 0.413	0.814
Wild type	32 (59.3)	14 (66.7)		
Mutation type	18 (33.3)	6 (28.6)		
Unknown	4 (7.4)	1 (4.8)		
NACT, n (%)			χ2 = 0.090	0.764
Yes	20 (37.0)	7 (33.3)		
No	34 (63.0)	14 (66.7)		
The residual disease			χ2 = 4.175	0.243
R0	18 (33.3)	11 (52.4)		
R1	21 (38.9)	6 (28.7)		
R2	5 (9.3)	3 (14.3)		
Unknown	10 (18.5)	1 (4.8)		
Histology, n (%)			χ2 = 0.089	0.765
Serous	50 (92.6)	19 (92.0)		
Others	4 (7.4)	2 (9.5)		
Previous lines of chemotherapy, n (%)			χ2 = 0.009	0.924
2	38 (70.4)	15 (71.4)		
3	13 (24.1)	4 (19.0)		
≥4	3 (5.6)	2 (9.5)		
PFI, n (%)			χ2 = 0.216	0.642
6-12 months	20 (37.0)	9 (42.9)		
>12 months	34 (63.0)	12 (57.1)		
Response to the last platinum-based therapy, n (%)			χ2 = 1.881	0.170
CR	22 (40.7)	5 (23.8)		
PR	32 (59.3)	16 (76.2)		
SCR, n (%)			χ2 = 0.000	0.987
Yes	7 (13.0)	2 (9.5)		
No	47 (87.0)	19 (90.5)		
The interval between the last chemotherapy and maintenance therapy, n (%)			χ2 = 1.498	0.221
4-8 weeks	34 (63.0)	17 (81.0)		
>8 weeks	20 (37.0)	4 (19.0)		
Combined with bevacizumab in maintenance therapy, n (%)			χ2 = 0.029	0.865
Yes	5 (9.3)	1 (4.8)		
No	49 (90.7)	20 (95.2)		
CA125 before PARPi, n (%)			χ2 = 0.750	0.386
<35U/ml	53 (98.1)	19 (90.5)		
≥35U/ml	1 (1.9)	2 (9.5)		
Time of PARPi treatment, median (Q1,Q3)	14 (8-21)	6 (4.5-15)	–	0.056
PARPi, n (%)				
Dose reduction	21 (38.9)	4 (19.0)	χ2 = 2.679	0.102
Dose interruption	13 (24.1)	9 (42.9)	χ2 = 2.573	0.109
Dose discontinuation	0 (0)	2 (9.5)	–	0.157

### 3.2 Efficacy

**Figure 2:**
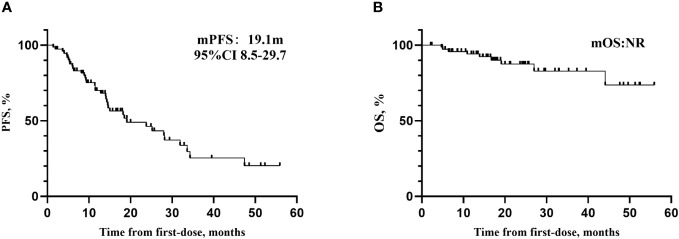
**(A)** Kaplan–Meier curves for PFS. **(B)** Kaplan–Meier curves for OS.

Out of the 75 patients diagnosed with PSROC, the median follow-up time was 20.0 months (95% CI 11.5-28.6). Among these patients, 38 experienced disease progression, and 9 died. The mPFS was 19.1 months (95% CI 8.5-29.7), while the mOS has not been reached yet (**Figure 2**). In the group receiving olaparib, the mPFS was 19.1 months, while in the group receiving niraparib, the mPFS was 28.2 months.

### 3.3 Influencing factors for PFS

A Log-rank univariate analysis was conducted to identify factors influencing PFS in patients with PSROC. It was found that age and the number of prior lines of chemotherapy were significantly associated with PFS (*P*<0.05). These factors with a significance level of P<0.05 were included in the multivariate Cox regression analysis. However, the results of the multivariate analysis indicated that neither age nor the number of prior lines of chemotherapy were independent factors influencing PFS in patients with PSROC (*P*>0.05). More detailed information can be found in **Tables 2**, **3**.

**Table 2:** Log-rank analysis of factors associated with PFS.

Clinical characteristics		Log-Rank analysis		
		mPFS (95%CI)	χ^2^	*P*
Age	<65	25.2 (13.0-37.4)	4.701	0.030*
	≥ 65	8.9 (2.3-15.4)		
Complication	Yes	18.5 (12.4-24.6)	0.399	0.527
	No	23.8 (9.2-38.3)		
Family history	Yes	28.0 (8.3-47.7)	1.540	0.215
	No	18.2 (12.1-24.3)		
BRCA gene	Wild type	23.8 (6.7-40.8)	0.052	0.974
	Mutation type	18.2 (12.2-24.2)		
	Unknown	NE		
NACT	Yes	14.0 (3.9-24.1)	2.992	0.084
	No	28.0 (10.3-45.7)		
The residual disease	≤R1	28.2 (13.2-43.2)	4.161	0.125
	R2	8.9 (0.1-17.7)		
	unknown	18.2 (11.9-24.5)		
Previous lines of chemotherapy	2	28.0 (14.7-41.3)	7.241	0.027*
	3	23.8 (9.9-37.6)		
	≥4	5.3 (.0-12.7)		
Response to the last platinum-based therapy	CR	25.2 (10.7-39.8)	1.172	0.279
	PR	14.6 (9.7-19.6)		
PFI	6-12months	18.5 (3.8-33.1)	0.291	0.589
	>12months	19.1 (1.7-36.6)		
SCR	Yes	31.9 (NE)	0.482	0.487
	No	19.1 (7.3-30.9)		
The interval between the last chemotherapy and maintenance therapy	4-8 weeks	23.8 (11.1-36.4)	0.067	0.795
	>8weeks	19.1 (1.5-36.8)		
The type of PARPi	Olaparib	19.1 (8.7-29.6)	0.000	0.986
	Niraparib	28.2 (0.3-56.1)		
Combined with bevacizumab in maintenance therapy	Yes	NE	0.020	0.887
	No	19.1 (8.3-30.0)		
PARPi interruption	Yes	15.0 (11.5-18.5)	0.109	0.741
	No	25.2 (12.0-38.4)		
PARPi reduction	Yes	47.4 (5.8-89.0)	3.618	0.057
	No	18.5 (12.2-24.8)		

* The factor with a significance level of P<0.05 was included in the multivariate analysis.

**Table 3:** COX analysis of factors associated with PFS.

Clinical characteristics	COX analysis							
	B	SE	Wald	df	Sig.	Exp(B)	95.0% CI	
							Lower	Upper
Age(<65y V.S.≥65y)	0.678	0.540	1.572	1	0.210	1.969	0.683	5.680
Previous lines of chemotherapy			3.126	2	0.209			
2 V.S. 3	0.284	0.387	0.538	1	0.463	1.328	0.622	2.834
2 V.S. ≥4	0.966	0.573	2.842	1	0.092	2.628	0.855	8.085

### 3.4 Safety for PARPi in the real world

The safety of PARPi in real-world clinical practice was evaluated in two groups, olaparib and niraparib (refer to **Table 4**). In the olaparib group (N=54), the most ccommon AEs included leukopenia (30/54, 40.0%), anemia (26/54, 34.7%), vomiting (24/54, 32.0%), and thrombocytopenia (21/54, 28.0%). The most common grade ≥3 AEs were anemia (8/54, 10.7%), thrombocytopenia (4/54, 5.3%), and leukopenia (1/54, 1.3%). In niraparib group (N=21), anemia (10/21, 47.6%), vomiting (10/21, 47.6%), leukopenia (9/21, 42.9%), and nausea (9/21, 42.9%) were the most common AEs. Moreover, grade ≥3 AEs included anemia (4/21, 19.0%) and thrombocytopenia (1/21, 4.8%). Notably, no MDS/AML events or new primary malignant tumors were reported by the end of the study. Furthermore, no additional safety signals were identified.

**Table 4:** Common AEs for olaparib and niraparib in the real world.

Terms	Olaparib(N=54)		Niraparib(N=21)	
	N(%)	≥G3(%)	N(%)	≥G3(%)
Hematological system				
Anemia	26(34.7)	8(10.7)	10(47.6)	4(19.0)
Leukopenia	30(40.0)	1(1.3)	9(42.9)	0
Thrombocytopenia	21(28.0)	4(5.3)	5(23.8)	1(4.8)
Gastrointestinal system				
Nausea	22(29.3)	0	9(42.9)	0
Vomiting	24(32.0)	0	10(47.6)	0
Diarrhea	4(5.3)	0	2(9.5)	0
Constipation	10(13.3)	0	3(14.3)	0
Abdominal pain	0	0	0	0
Loss of appetite	20(26.7)	0	6(28.6)	0
Fatigue	10(13.3)	0	6(28.6)	0
Infection and invasive disease				
Upper respiratory tract infection	6(8.0)	0	1(4.8)	0
Urinary tract infection	2(2.7)	0	4(19.0)	0
Pneumonia	1(1.3)	0	0	0
Neurological System				
Dizziness/Headache	0	0	0	0
Sleeping disorders	13(17.3)	0	4(19.0)	0
Cardiovascular System				
Tachycardia	5(6.7)	0	4(19.0)	0
Hypertension	0	0	2(9.5)	0
Abdominal liver and kidney function				
Elevated transaminases	6(8.0)	0	5(23.8)	0
Elevated creatinine	14(18.7)	0	5(23.8)	0
Kidney failure	0	0	0	0
Others				
Muscle, skeletal and joint pain	10(13.3)	0	1(4.8)	0
Dermatitis, rash, photosensitivity	3(4.0)	0	3(14.3)	0
Oral ulcers, oral mucositis	8(10.7)	0	1(4.8)	0

In this single-center real-world study, approximately 29.3% of patients (22/75) interrupted treatment, 13.3% (10/75) of whom interrupted the medication due to grade ≥3 AEs, and all 10 cases were anemia (see **Table 5**). Moreover, 33.3% of patients (25/75) experienced dose reduction, 20.0% (15/75) of which were associated with hematological AEs. Specifically, 5.3% (4/75) underwent dose reduction due to grade≥3 AEs, including anemia (3/75, 4.0%) and thrombocytopenia (1/75, 1.3%). Notably, no patients discontinued PARPi treatment due to AEs.

**Table 5:** Common AEs for PARPi Interruption, Reduction, and Discontinuation.

Terms	Dose interruption		Dose reduction		Dose discontinuation	
	N(%)	≥G3(%)	N(%)	≥G3(%)	N(%)	≥G3(%)
	22(29.3)	10(13.3)	25(33.3)	4(5.3)	0	0
Hematological system						
Anemia	10(13.3)	10(13.3)	5(6.7)	3(4.0)	0	0
Leukopenia	2(2.7)	0	4(5.3)	0	0	0
Thrombocytopenia	5(6.7)	0	2(2.7)	0	0	0
Bone marrow suppression	1(1.3)	0	4(5.3)	1(1.3)	0	0
Gastrointestinal system						
Nausea	0	0	3(4.0)	0	0	0
Vomiting	1(1.3)	0	0	0	0	0
Diarrhea	0	0	0	0	0	0
Constipation	0	0	0	0	0	0
Abdominal pain	0	0	0	0	0	0
Loss of appetite	1(1.3)	0	2(2.7)	0	0	0
Fatigue	0	0	0	0	0	0
Infection and invasive disease						
Upper respiratory tract infection	0	0	0	0	0	0
Urinary tract infection	1(1.3)	0	0	0	0	0
Neurological System						
Dizziness/Headache	0	0	0	0	0	0
Sleeping disorders	0	0	0	0	0	0
Cardiovascular System						
Tachycardia	0	0	0	0	0	0
Hypertension	0	0	0	0	0	0
Abdominal liver and kidney function						
Elevated transaminases	0	0	0	0	0	0
Elevated creatinine	1(1.3)	0	5(6.7)	0	0	0
Kidney failure	0	0	0	0	0	0

## 4 Discussion

PARPi has become the standard treatment for maintenance therapy in patients with PSROC, as it has successfully broken two “70%” barriers for ovarian cancer patients (4, 5). This validation was achieved through multiple large-scale Phase III RCTs. However, the complexity of the relationship between patients and doctors in the clinical practice exceeds that observed in RCTs. The relationship between healthcare providers and patients has evolved from the traditional model of passive-active and guidance-cooperation to a new model of shared participation. This model involves joint consultation to make individualized diagnosis and treatment decisions tailored to the patient’s condition, while also considering the patient’s preferences and economic status. Thus, the evaluation of the efficacy and safety of PARPi in real-world clinical settings, alongside the findings of large-scale RCT studies, offers enhanced external validity. This study, conducted using real-world clinical data at the largest gynecologic oncology center in Western China, reaffirmed the effectiveness and favorable tolerability of PARPi in patients with PSROC. Moreover, it contributed significantly to the growing body of knowledge on maintenance therapy for ovarian cancer and offered valuable insights for the clinical implementation of PARPi.

The initial strategy for managing PSROC focuses on prolonging the time to recurrence and diminishing the likelihood of recurrence (18). According to the 2023 version of the National Comprehensive Cancer Network(NCCN) Ovarian Cancer Guidelines, patients with PSROC who have achieved CR or PR after last platinum-based chemotherapy, and have not received PARPi before, were advised to undergo maintenance therapy with PARP inhibitors. This guideline recommends Olaparib for all PSROC patients, irrespective of their BRCA status, while limiting the use of niraparib to gBRCAm patients and rucaparib to BRCAm patients (19). The 2022 American Society of Clinical Oncology (ASCO) guidelines recommend PARPi monotherapy as a maintenance therapy after platinum-sensitive recurrence, regardless of the BRCA mutation status (20). Many studies have demonstrated the efficacy of PARPi monotherapy in PSROC patients who previously received ≥2nd-line of platinum-based chemotherapy. The mPFS was 8.4 months in Study19 (N=136, olaparib) (21), 16.1 months in L-MOCA study (N=224, olaparib) (8), 15 months in NORA study (N=177, niraparib) (9), and 12.9 months in FZOCUS-2 study (N =167, fluzoparib) (22). In this real-world study, the mPFS of overall population was 19.1 months (95%CI 8.5-29.7). Specifically, the mPFS for the olaparib and niraparib groups were 19.1 months and 28.2 months, respectively. It is noteworthy that in our center, both in the overall population and in the olaparib or niraparib group, the mPFS was longer than that in the previously mentioned large clinical trials. The follow-up time may be the reason for this difference. From the published data so far, the median follow-up time is 6.9 months (206.5 days) in Study19 (21), 15.5 months in L-MOCA study (8), 15.8 months in NORA study (9), and 8.5 months in FZOCUS-2 study (22). Notably, the follow-up time in our center was longer at 20.0 months (95%CI 11.5-28.6), which might be a reason for this difference. According to a domestic study on 106 PSROC patients, with a median follow-up of 17.5 months (95% CI 13-22), 49 patients had received PARPi for at least 12 months at the time of analysis. The mPFS from the initiation of PARPi was 21 months (95% CI 13–24.5) (23). In another study conducted in China, 48 PSROC patients who achieved CR or PR after last platinum-based chemotherapy were included. This study reported a median follow-up time of 17.8 months and a mPFS of 26.1 months (95% CI 20.2-32.1) (24). Hence, the patients with PSROC in the Chinese population experienced significant PFS benefits from PARPi. However, as the studies were non-head-to-head and the RWE was limited, the results can only be considered as a reference.

SCR in patients with PSROC is controversial (25). Whether SCR will affect the efficacy of PARPi is worth exploring. A non-randomized case-control study (26) included 46 patients with PSROC carrying BRCA-m. The case group received SCR + chemotherapy + olaparib (N=23), and the control group received chemotherapy + olaparib (N=23), the baseline data of the two groups were well balanced and comparable. The case group exhibited a significantly longer median duration of subsequent treatment compared to the control group (42 months versus 16 months, *P* =0.05). Furthermore, the 3-year survival rate after recurrence was significantly higher in the case group than in the control group (79% V.S. 42%, *P* =0.02). A RWS in China included 106 PSROC patients with 19 patients (17.9%) receiving SCR after relapse. COX regression analysis indicated that receiving SCR was not significantly associated with prolonged PFS (HR=0.88, 95% CI 0.38-2.00, *P*=0.761). A phase II RCT (27) (NCT03983226) is currently underway to investigate the potential benefits of niraparib for PSR ovarian cancer patients undergoing SCR. In this study, among the patients with PSROC and achieving CR or PR after the last chemotherapy, 9 cases underwent SCR after recurrence. The SCR + chemotherapy group (N=9) demonstrated a prolongation of mPFS by 12.8 months (31.9 months V.S. 19.1 months) compared to the chemotherapy-only group (N=57). This outcome suggested a potential benefit of adding SCR to chemotherapy for PSR ovarian cancer patients. However, further evidence from high-quality clinical trials is needed to determine whether the use of PARPi would weaken the effect of SCR.

PFI is used to measure the sensitivity of platinum-based drugs. In the L-MOCA study conducted on the Asian-Pacific Chinese population (8), the mPFS in the complete platinum-sensitive group (N=70) was 20.9 months (95% CI 16.2-24.1), while that in the partial platinum-sensitive group (N=67) was 9.3 months (95% CI 8.3-14.1). Patients with a PFI >12 months showed a potential trend towards benefit. However, in the NORA study (9), the risk of disease progression or death who taking niraparib was reduced by 69% (95%CI 0.17-0.55) and 67% (95% CI 0.22-0.51) in the partial platinum-sensitive group and the complete platinum-sensitive group. Interestingly, the study revealed that PFI>12 months did not have a significant impact on PFS in patients. Additionally, the forest plot presented in Study 19 (21) did not show a significant difference in PFS between partial platinum-sensitive and complete platinum-sensitive patients. A European multi-center retrospective study (28) included 114 patients with recurrent ovarian cancer carrying BRCA mutation. In patients with a PFI<12 months(N=40), the mPFS was 10.4 months (95% CI 6.3-17.1), while in patients with a PFI≥12 months (N=74), the mPFS was 18.0 months (95% CI 10.1-26.8). Compared to patients with a PFI<12 months, patients with a PFI≥12 months showed a significant extension in PFS (HR=0.5, 95% CI 0.6-0.8, *P*<0.01). In this study, the mPFS was 18.5m for patients with PFI of 6-12 months, and 19.1 months for patients with PFI>12 months, and the difference was not statistically significant (Log-rank, χ2 = 0.291, *P*=0.589). Further exploration is needed to determine whether the degree of platinum sensitivity affects the efficacy of PARPi in PSROC patients.

In this RWS, the mPFS of BRCA-m group (N=24) and BRCA-wt group (N=46) were 18.2 months and 23.8 months, respectively. There was no statistically significant difference in PFS between patients (Log-rank, χ2 = 0.052, *P*=0.974). The reasons for the analysis are as follows: (1) The proportion of partially platinum-sensitive patients in the BRCA-m group was found to be higher than that in the BRCA-wt group (45.8% V.S. 34.8%). (2) This observation may be attributed to factors such as the relatively small sample size of the subgroup and data immaturity. In addition, it is pertinent to note that 5 patients in our center declined genetic testing due to economic constraints. Nevertheless, it is noteworthy that the completion rate of BRCA gene testing reached 93.3%. This high completion rate underscored our center’s strict adherence to diagnostic and treatment guidelines, as well as our commitment to patient education. The Society of Gynecologic Oncology (SGO) in 2021 released the latest data from the NOVA study (29, 30), indicating that non-BRCAm patients in the niraparib group exhibited a 5.4-month reduction in mOS compared to the control group (31.1 months V.S. 36.5 months).This suggests that niraparib maintenance therapy in patients without BRCA-m may be associated with a detrimental effect on OS (HR=1.10, 95% CI 0.831-1.459). Hence, it is evident that while patients without BRCA-m may potentially benefit from niraparib in terms of PFS, there is a decreasing trend in OS. Consequently, the first version of the NCCN guidelines (19) in 2023 was revised to specify that niraparib is restricted to gBRCAm patients. Furthermore, the 2022 ASCO meeting highlighted the need to carefully consider the balance between potential PFS benefits and OS decline when utilizing niraparib maintenance therapy for patients with non-BRCA mutations (20). The NORA study, which focused on the Chinese population and utilized individualized starting doses, demonstrated OS benefits for the entire population receiving niraparib as maintenance therapy, regardless of BRCA gene status after applying inverse probability weighting (10). The survival differences between the NOVA and NORA studies are summarized in **Table 6**. This suggested that the individualized starting doses used in the NORA study may have contributed to the observed OS benefits, which is an important consideration when evaluating the efficacy of niraparib as a maintenance therapy. In conclusion, it is currently uncertain whether the NCCN guidelines will impose more stringent restrictions on the utilization of PARP inhibitors in the PSROC population. Nevertheless, irrespective of the guidelines, healthcare professionals should prioritize patient education, emphasize the importance of genetic test in genetic assessment, efficacy, and prognosis, and promote patients’ willingness to undergo HRD testing. Additionally, after examining studies including NOVA study (30), SOLO-2 study (7), and Study 19 (21), it is evident that there is a limited representation of Chinese patients in these global clinical trials. The clinical trial evidence for PARP inhibitors approved by the U.S. Food and Drug Administration (FDA) may differ from that approved by the China National Medical Products Administration (NMPA). We look forward to the development of more multi-center clinical studies conducted on Chinese and Asian populations.

**Table 6:** The survival comparison between NOVA and NORA.

Study	NOVA (30)				NORA (10)			
Groups	Niraparib	Placebo	HR	95%CI	Niraparib	Placebo	HR	95%CI
BRCA-m	43.5	41.6	0.93	0.63-1.34	NR	42.1	0.88	0.39-2.01
Non-BRCAm	31.1	36.5	1.10	0.83-1.46	43.1	32.6	0.62	0.37-1.51
ITT*	38.5	39.1	N/A	N/A	46.3	34.3	0.69	0.45-1.07

*ITT, intention-to-treat population. N/A,not available.

The majority of advanced ovarian cancer patients experience recurrent or progressive disease, leading to a gradual shortening of the PFI after multiple lines of chemotherapy, and ultimately developing drug resistance. Therefore, in our study, we conducted a subgroup analysis based on the number of prior lines of chemotherapy. An analysis of the data showed that 70.4% of patients had received 2^nd^-line chemotherapy, 24.1% had received 3^rd^-line chemotherapy, and 5.6% had received more than 4^th^-line chemotherapy. Furthermore, a trend was revealed wherein patients receiving 2^nd^-line chemotherapy exhibited a potentially enhanced PFS compared to those who had undergone 3^rd^-line or more than 4th-line chemotherapies. (2^nd^-line V.S. 3^rd^-line V.S. ≥ 4^th^-line: 28.0 months V.S. 23.2 months V.S. 5.3 months). In the L-MOCA study (23), patients who previously received 2^nd^-line chemotherapy demonstrated a mPFS of 9.2 months longer than patients who previously received 3^rd^-line chemotherapy (18.0 months V.S. 8.8 months). In a RWS involving 234 PSROC patients with BRCA-m treated with olaparib (31), the median follow-up time was 15.5 months (95% CI 13.0-18.2). Patients who had received 2^nd^-line chemotherapy had a longer PFS than those who had received 3^rd^-line or more chemotherapy, with mPFS of 16.6 months, 15.5 months, and 8.2 months, respectively (2^nd^-line V.S. 3^rd^-line: HR=1.9, 95% CI 1.1-3.6, *P*=0.03; 2^nd^-line V.S. 3^rd^-line: HR=2.5, 95% CI 1.34-4.8, *P*=0.004). It is expected to increase the sample size, lengthen the follow-up time, and further explore the impact of the number of prior lines of chemotherapy on the prognosis of patients.

In this study, the maturity of PFS data in the PSR population was 50.7% (38/75). Among patients who achieved CR and PR after the last chemotherapy, the mPFS was 25.2 months (95%CI 10.7-39.8) and 14.6 months (95%CI 9.7-39.8). Notably, the mPFS of the CR group was 10.6 months longer than that of the PR group. However, a statistical analysis revealed no significant difference in PFS between the CR and PR groups (Log-rank, χ2 = 1.172, *P*=0.279), which could be attributed to the sample size and follow-up time. In the subgroup analysis of the SOLO2 study (32), the mPFS of patients who achieved CR at the last chemotherapy (N=91) has not yet reached, while the mPFS of the PR group (N=105) was 13.8 months. The risk of disease progression or death was reduced by 74% (HR=0.26, 95%CI 0.16-0.42) and 63% (HR=0.37, 95%CI 0.25-0.54), respectively. At the same time, the CR group showed a significant benefit compared with the PR group. This finding aligned with the results of the NORA study (9), wherein similar trends were observed. In the NORA study (9), patients in the CR and PR groups experienced a reduction in the risk of disease progression or death by 74% (95%CI 0.15-0.45) and 67% (95%CI 0.21-0.52), respectively. In the L-MOCA study (8), the mPFS for the CR group (N=43) was 19.7 months (95%CI 15.8-22.2), while that of the PR group was 13.9 months (95%CI 11.0-16.6). The mPFS of the CR group was 5.8 months longer than that of the PR group. According to Study19 (21), the risk of disease progression or death in CR patients decreased by 54% (HR=0.46, *P*<0.001). Additionally, several real-world studies based on the Chinese population also found that achieving CR after the last chemotherapy was associated with improved PFS (31, 33). After 15.5 months (95%CI 13.0-18.2) follow-up, a study of 234 PSROC patients with BRCA-m treated with olaparib showed that the mPFS for patients who achieved CR, PR, SD and PD after last chemotherapy was 33.4 months, 10.4 months, and 9.2 months, respectively (CR V.S. PR: HR=3.1, 95% CI 1.6-5.8, *P*=0.001; CR V.S. SD+PD: HR=2.7, 95% CI 1.2-6.1, *P*=0.017). This indicates that patients who achieved CR had significant PFS benefit compared to those with PR or SD+PD. (CR V.S. PR: HR=3.1, 95%CI 1.6-5.8, *P*=0.001; CR V.S. SD+PD: HR=2.7, 95%CI 1.2-6.1, *P*=0.017) (31). That is, patients who achieved CR with the last chemotherapy had a prolonged PFS compared with patients with PR or SD+PD. In a RWS with a total of 106 patients, 47 patients achieved CR and 59 patients achieved PR, with a median follow-up time of 17.5 months (95% CI: 13-22) (23). The study revealed that achieving CR after the last chemotherapy was an independent factor influencing PFS in patients with PSROC (HR=0.42, 95% CI 0.21-0.85, *P*=0.016). This finding was supported by another RWS of olaparib in 97 patients with PSROC, which found that after 13 months of follow-up, the risk of disease progression or death in patients who achieved CR decreased by 58.6% (HR=0.414, 95% CI 0.205 -0.836, *P*=0.014) (33). Therefore, it is evident that the achievement of CR following the last chemotherapy has a substantial impact on PFS in patients with PSROC.

Maintenance therapy aims to achieve long-term disease control, and the timely identification, continuous monitoring, and effective management of AEs directly influence patient compliance with the medication, thereby impacting treatment efficacy. PARP inhibitor-related AEs commonly occur within the first three months of treatment and are primary reasons for patients to adjust the drug dosage, interrupt treatment, or even discontinue the medication (34, 35). Based on the real-world safety data of patients in our hospital receiving PARP inhibitors (PARPi), this study found that the most common adverse events (AEs) were hematologic toxicity and gastrointestinal reactions. Hematologic toxicity was the most common grade ≥3 AE. This may be attributed to the fact that PARPi can target PARP enzymes involved in various physiological processes, including the regulation of cell differentiation in the bone marrow or hematopoietic system by PARP1, and the involvement of PARP2 in the regulation of red blood cell production (36, 37). Different PARP inhibitors can cause varying hematologic toxicities (34, 35). In our center, the population receiving olaparib, the incidence of grade ≥3 anemia was 10.7% (8/54). In the SOLO-1 study (5), the incidence of grade ≥3 anemia was 22%, while in the SOLO-2 study (7), it was 19%. The incidence of grade ≥3 leukopenia was 1.3% (1/54) in olaparib group in this RWS, compared to 2% in the SOLO-2 study (7). The incidence of grade≥3 thrombocytopenia in olaparib group was 5.3% (4/54), while in the SOLO-1 study (5) and SOLO-2 study (7) the incidence of grade≥3 thrombocytopenia was 1%. The incidence of hematological AEs of olaparib is basically consistent with the data of large clinical trials. In this study, 4 patients taking niraparib had grade ≥ 3 anemia (4/21, 19.0%), and 1 patient had grade≥3 thrombocytopenia (1/21, 4.8%). In the PRIMA study (38) and NOVA study (30), the incidence rates of grade ≥3 anemia were 31% and 25%, and the incidence rates of grade ≥3 thrombocytopenia were 29% and 34%, respectively. Compared with the data of large clinical trials, the incidence of hematological in niraparib group in our center is relatively low. This may be related to the individualized starting dose administration, and the rigorous monitoring and management of complete blood counts. Notably, none of the patients taking niraparib discontinued treatment due to thrombocytopenia at the end of the follow-up period, in contrast to 4% reported in the NORA study (9, 10) and 14.7% in the NOVA study (30).

MDS/AML is a delayed adverse event associated with PARPi therapy. Morice et al. (39) conducted a meta-analysis of 28 randomized controlled trials (RCTs) of PARP inhibitors published between March 2012 and April 2020 to assess the occurrence of MDS/AML associated with PARPi and found that all cases of MDS/AML were observed in ovarian cancer patients. The World Health Organization’s VigiBase database from 2015 to 2020 revealed a total of 178 cases of MDS/AML associated with the use of PARPi. Among these cases, 58 patients developed MDS/AML after their initial use of PARPi, with a median latency period of 17.8 months (39, 40). In the SOLO2 study (7), there were 4 cases of MDS/AML in the olaparib group. In the NOVA study (29, 30), the niraparib group had 5 cases of MDS/AML. In the PAOLA-1 study (41), the olaparib + bevacizumab group had 5 cases of MDS/AML. In the ARIEL-3 study (42), the rucaparib group had 4 cases of MDS/AML. As of the follow-up endpoint, no cases of MDS/AML have been observed in patients receiving olaparib or niraparib in our center. However, as a delayed AE, it is necessary to be vigilant against MDS/AML in the follow-up time. Once it occurs, the treatment should be stopped immediately and go to the hematology department (35). Currently, there is a lack of real-world clinical studies on the safety monitoring for various PARP inhibitors in China. We look forward to more research in this area.

## 5 Limitation

Due to the small sample size, this single-center real-world clinical study has limitations in performing more specific subgroup analyses. Additionally, the insufficient data maturity, particularly with regards to the OS data, demands long-term follow-up to analyze the factors influencing the clinical benefit of PARPi. Additionally, this study primarily relied on data collection from the West China Second University Hospital’s Hospital Information System (HIS), and it was retrospective in nature, which led to some limitations in the collection of safety data, such as the exact time when the AEs started and stopped during the PARPi period, the investigator’s assessment of the causal relationship between the AEs and PARPi, the assessment of the causal relationship with other medications, the treatment measures taken for the AEs, and the outcome. Further standardization is needed in the collection and administration of safety data. The 2023 NCCN guidelines recommend explicitly determining the HRD status. For BRCA-wt patients, testing for HRD can improve patient prognosis. However, our center is located in the western China with relatively less-developed economy. Most patients were unable to complete HRD testing due to the high cost of HRD testing and the limited availability of HRD testing kits in the domestic market, especially for those who have completed BRCA testing. Therefore, this study did not analyze HRD-related data.

## 6 Conclusion

This real-world study confirmed the efficacy and safety of olaparib and niraparib in the treatment of PSROC. No MDS/AML was observed in this study. However, it remains necessary to exercise close follow-up to remain vigilant for the occurrence of secondary tumors. The importance of genetic testing is emphasized, and it is encouraged to improve HRD testing for non-BRCAm patients to guide treatment and improve patient prognosis.

## Data availability statement

The original contributions presented in the study are included in the article/supplementary material. Further inquiries can be directed to the corresponding author.

## Ethics statement

This study followed the Declaration of Helsinki and was approved by the Ethics Committee of West China Second Hospital of Sichuan University (approval number: 20220129). The studies were conducted in accordance with the local legislation and institutional requirements. Written informed consent for participation was not required from the participants or the participants’ legal guardians/next of kin because Informed consent was waived by competent authorities due to the anonymized nature of patient data and the retrospective design of the study.

## Author contributions

RY: Conceptualization, Funding acquisition, Project administration, Resources, Supervision, Validation, Writing – review & editing. JC: Data curation, Formal analysis, Methodology, Software, Writing – original draft. MZ: Data curation, Investigation, Writing – original draft. KL: Formal analysis, Methodology, Software, Writing – original draft. YD: Data curation, Writing – original draft. XL: Validation, Writing – original draft. LZ: Validation, Writing – original draft. QL: Supervision, Funding acquisition, Writing – review & editing.
